# Genetic ancestry and radical prostatectomy findings in Hispanic/Latino patients

**DOI:** 10.3389/fonc.2024.1338250

**Published:** 2024-04-03

**Authors:** Natalia L. Acosta-Vega, Rodolfo Varela, Jorge Andrés Mesa, Jone Garai, Alberto Gómez-Gutiérrez, Silvia J. Serrano-Gómez, Jovanny Zabaleta, María Carolina Sanabria-Salas, Alba L. Combita

**Affiliations:** ^1^ Grupo de Investigación en Biología del Cáncer, Instituto Nacional de Cancerología de Colombia, Bogotá D.C., Colombia; ^2^ Programa de doctorado en Ciencias Biológicas, Pontificia Universidad Javeriana, Bogotá D.C., Colombia; ^3^ Departamento de Urología, Instituto Nacional de Cancerología de Colombia, Bogotá D.C., Colombia; ^4^ Departamento de Cirugía, Facultad de Medicina, Universidad Nacional de Colombia, Bogotá D.C., Colombia; ^5^ Departamento de Patología Oncológica, Instituto Nacional de Cancerología de Colombia, Bogotá D.C., Colombia; ^6^ Stanley S. Scott Cancer Center, Louisiana State University Health Sciences Center, New Orleans, LA, United States; ^7^ Instituto de Genética Humana, Facultad de Medicina, Pontificia Universidad Javeriana, Bogotá D.C., Colombia; ^8^ Department of Interdisciplinary Oncology, School of Medicine, Louisiana State University Health Sciences Center, New Orleans, LA, United States; ^9^ Departamento de Microbiología, Facultad de Medicina, Universidad Nacional de Colombia, Bogotá D.C., Colombia

**Keywords:** prostatic neoplasms, Gleason Grade Groups (GG), genetic ancestry, biochemical recurrence, Hispanic/Latino population

## Abstract

**Background:**

African ancestry is a known factor associated with the presentation and aggressiveness of prostate cancer (PC). Hispanic/Latino populations exhibit varying degrees of genetic admixture across Latin American countries, leading to diverse levels of African ancestry. However, it remains unclear whether genetic ancestry plays a role in the aggressiveness of PC in Hispanic/Latino patients. We explored the associations between genetic ancestry and the clinicopathological data in Hispanic/Latino PC patients from Colombia.

**Patients and methods:**

We estimated the European, Indigenous and African genetic ancestry, of 230 Colombian patients with localized/regionally advanced PC through a validated panel for genotypification of 106 Ancestry Informative Markers. We examined the associations of the genetic ancestry components with the Gleason Grade Groups (GG) and the clinicopathological characteristics.

**Results:**

No association was observed between the genetic ancestry with the biochemical recurrence or Gleason GG; however, in a two groups comparison, there were statistically significant differences between GG3 and GG4/GG5 for European ancestry, with a higher mean ancestry proportion in GG4/GG5. A lower risk of being diagnosed at an advanced age was observed for patients with high African ancestry than those with low African ancestry patients (OR: 0.96, CI: 0.92-0.99, p=0.03).

**Conclusion:**

Our findings revealed an increased risk of presentation of PC at an earlier age in patients with higher African ancestry compared to patients with lower African ancestry in our Hispanic/Latino patients.

## 1 Introduction

Prostate cancer (PC) is the second most common cancer and the fifth leading cause of death from cancer in men worldwide (1). In Colombia, PC is the most common cancer in men with estimated age-standardized incidence rates of 49.8 cases per 100,000 inhabitants and second highest mortality rates with 12-12.6 per 100,000 inhabitants (2, 3).

The progression from localized PC to metastatic disease can vary considerably from one patient to another, most of them show progression over many decades, while others show an aggressive and rapidly progressive disease that can lead to death (4). These differences may be attributable to multiple factors including age (5), socioeconomic factors (6), access to healthcare (7), lifestyle factors and genetic differences within a population that can modulate the prognosis of the disease or the molecular heterogeneity of tumors (8, 9). For Hispanic populations, the situation is not different, in which factors such as obesity, smoking, low physical activity levels, lack of access to high-quality care and lower socioeconomic status can further contribute to disproportionately impact these communities; and play an essential role in mediating PC disparities (10, 11). On the other hand, PC is a multifocal pathology, in which a single prostate may harbor multiple genetically distinct tumor foci that may be indistinguishable by routine histology (12).

The histological Gleason grading has been one of the most important predictors of biochemical recurrence, disease progression and survival, and is related with response to different therapies (13, 14). This grading system, introduced by Donald F. Gleason in 1996 (15), was recently updated and modified to a new system with Gleason Grade Groups (GG) from 1 to 5 (16). This was done in order to decrease some of the limitations associated with the interobserver variability (17, 18) since the percentage of Gleason pattern four in the sample is a key factor associated with a different risk depending on whether it is the most prevalent or not (19). With the new grading system, GG4 and GG5 harbor higher risks and correlate with progression to metastasis, while GG1 carries a lower risk (20–22), and even have been reported not to progress within a 10-year follow-up and hence being possible candidates for management with active surveillance (23).

Genetic ancestry has been one of the risk factors associated with PC, in which a higher incidence and mortality, as well as more aggressive tumors, have been observed in men with African ancestry compared to other ethnic groups (24). In 2006, the first locus on chromosome 8q24 conferring increasing risk for PC was identified (25). Other loci in the same region were later also found to be associated with increased risk, especially in men with African ancestry (26, 27). Another locus in 17q21 was also associated with increased risk of PC in African populations compared to Europeans and Asians (28), which led to consider African ancestry as a risk factor for the onset of PC, especially knowing that PC tumors develop into aggressive stages faster in African American individuals when compared to Europeans and Asians (29, 30).

The Colombian population is highly diverse and multiethnic due to the admixture of Native Americans, Europeans and West Africans (31). Afro-Colombians represent about 10.6% of the Colombian population according to self-reported ethnic identity from the 2005 Census and inhabit mainly the Coastal regions of the country. On the other hand, Indigenous communities represent 3.4% and are also mainly located in the southeast of the country, corresponding to the Plain region, and in the Coastal (National Administrative Department of Statistics – DANE) (32). However, the Mestizo group, which is the self-identification of White people or no ethnicity, reflects the individuals that are not considered to belong to any of the Colombian minority’s ethnic groups, and represents about 85% of the population. Some studies with Colombian patients demonstrated the effect of genetic ancestry on the risk of different types of cancer, including breast and colorectal cancer (33–35). It is still unknown if genetic ancestry plays a role in the aggressiveness of PC in Colombian patients, especially in a population with high incidence and mortality rates. This study explores the associations between genetic ancestry and the clinicopathological features in Colombian PC patients treated with radical prostatectomy.

## 2 Materials and methods

### 2.1 Patients and sample collection

PC patients treated with radical prostatectomy (RP) at Instituto Nacional de Cancerología (INC) in Colombia from 2007 to 2011 were included. Clinical information was obtained from the medical records at INC databases. The selection criteria included patients with no previous treatment other than RP, follow-up of at least one year after treatment and complete medical records to collect clinical information. BCR was defined as the elevation of serum PSA levels over 0.2 ng/mL on two successive measurements (36). For this study, the outcome of BCR was established within the 5 years of follow-up after RP surgery.

Samples from FFPE non-tumor tissues from RP specimens were used for genetic ancestry analysis. FFPE specimens from 289 cases were reviewed by expert pathologists to confirm Gleason GG and histopathological characteristics on these samples, as well as to select the non-tumor regions. For the genetic ancestry analysis, we utilized tissue arrayer needles to extract a total of four to seven tissue section cores, each measuring 0.6 mm in diameter. This study was approved by the Research Ethics Board at the INC and was designated as an exempt study for informed consent.

### 2.2 DNA extraction

DNA from adjacent non-tumoral tissue from 289 cases was extracted using AllPrep DNA/RNA FFPE kit^®^ (Qiagen, Hilden, Germany) following the manufacturer’s recommendations. DNA quantity and quality were determined with Nanodrop 2000 Spectrophotometer^®^ (ThermoFisher Scientific, Wilmington, USA), excluding 28 cases due to bad quality or quantity.

### 2.3 Ancestry estimation

DNA samples were sent to the University of Minnesota Genomics Center for the genotyping of 106 autosomal Ancestry-Informative Markers (AIMs) for the estimation of individual genetic ancestry (37), in a Sequenom iPLEX^®^ Genotyping Platform. Setting a call rate of 90% for the Single Nucleotide Polymorphisms (SNPs), 101 AIMS were suitable for ancestry estimation, and samples with a call rate under 85% excluded 31 samples from the analysis. All AIMs were in Hardy-Weinberg equilibrium. Estimation of proportions of genetic ancestry was performed as previously described (38). For each case, the proportions of European, African, and Indigenous American ancestry were estimated under an admixture model with the *ADMIXTURE* ^®^ software V1.3.0. A supervised analysis was performed with three parental reference populations, which were kindly provided by Dr. Laura Fejerman: European (42 individuals from Coriell’s North American Caucasian panel), African (37 non-admixed Africans living in the United Kingdom and South Carolina - USA), and Indigenous Central American populations (15 Mayan and 15 Nahuas) (37).

### 2.4 Statistical analysis

Genetic ancestry was modeled both as a continuous variable (percentage of European, Indigenous and African ancestry) and as a categorical variable (dichotomous for each ancestry population based on the median proportion). Sociodemographic and clinicopathological characteristics were evaluated according to the Gleason GG by applying analysis of variance test (ANOVA) and Kruskal-Wallis for continuous variables while categorical variables were analyzed for associations by *X^2^* test and Fisher’s exact test. The distribution of tumor characteristics was analyzed by categorized genetic ancestry component. When comparisons between two groups were assessed, the Student’s T-test and Wilcoxon rank-sum test were used. Logistic regression models were fitted to estimate the unadjusted and adjusted odds ratio (OR) and its 95% confidence interval (CI) for the association of Gleason GG, as well as clinicopathological characteristics, and genetic ancestry. All the statistical analyses were done in *Rstudio* ^®^ v1.1.463 and *p*-value < 0.05 was established for statistical significance.

## 3 Results

### 3.1 Description of the study population

We analyzed 230 cases after excluding 59 samples, as mentioned before. Demographics for these cases are shown in **Table 1**. Patients ranged from 32 to 75 years old at diagnosis with a median age of 64 years, most of the patients (65.9%, n=135) were diagnosed as overweight/obese (BMI ≥25). The median value of PSA at diagnosis was 8.65 ng/mL (IQR 6.21 – 13.49 ng/mL) and the distribution of these cases in the Gleason GG at biopsy accounted for 79.4% of cases across lower grades (GG1 and GG2); while the higher Gleason grades were less frequent at biopsy accounting only 20.6% (GG3 and GG4/GG5). The median time of follow-up was 60.18 months, with a maximum time being 10 years (120.83 months) and information for BCR in a 5-year follow-up was available for 110 out of the 230 cases (47.8%), with 39.1% (n=43) of cases positive. Finally, the distribution of cases for the Gleason GG at radical prostatectomies accounted for more than 60% across lower grades (GG1 and GG2), while the higher Gleason grades were less frequent, 32.6% (GG3 and GG4/GG5) (**Table 1**).

**Table 1:** Demographics and clinical characteristics of cases stratified by Gleason Grade groups at RP.

Characteristics	PC cohort	Gleason GG1	Gleason GG2	Gleason GG3	Gleason GG4/GG5	P value
	(n=230) n (%)	(n=80) n (%)	(n=75) n (%)	(n=60) n (%)	(n=15) n (%)	
**Age - years (median, IQR)**	64.0 (58.0-67.0)	64.0 (56.75- 68.0)	64.0 (58.0- 67.50)	63.5 (56.75-67.0)	63.0 (59.5-67.5)	0.922
**BMI (n = 205)**						0.064
** <25**	70 (34.1)	23 (31.5)	21 (31.8)	17 (32.7)	9 (64.3)	
**25-30**	107 (52.2)	40 (54.8)	40 (60.6)	23 (44.2)	4 (28.6)	
**>30**	28 (13.7)	10 (13.7)	5 (7.6)	12 (23.1)	1 (7.1)	
Pre-operative characteristic						
**Preoperative PSA (median, IQR)**	8.65 (6.21-13.49)	7.40 (5.31-10.36)	8.25 (6.40-13.83)	11.70 (7.35-15.62)	12.00 (9.91-22.90)	**<0.001**
**Gleason Grade Group at biopsy (*n = 199*)**						**<0.001**
**G1**	121 (60.8)	63 (92.6)	45 (69.2)	11 (20.8)	2 (15.4)	
**G2**	37 (18.6)	4 (5.9)	14 (21.5)	16 (30.2)	3 (23.1)	
**G3**	30 (15.1)	0 (0.0)	4 (6.2)	22 (41.5)	4 (30.8)	
**G4 and G5**	11 (5.5)	1 (1.5)	2 (3.1)	4 (7.5)	4 (30.8)	
**cT stage (*n = 227*)**						**0.006**
**cT_I**	89 (39.2)	36 (46.2)	33 (44.0)	19 (32.2)	1 (6.7)	
**cT_II**	136 (59.9)	42 (53.8)	41 (54.7)	40 (67.8)	13 (86.7)	
**cT_III**	1 (0.4)	0 (0.0)	1 (1.3)	0 (0.0)	0 (0.0)	
**cT_IV**	1 (0.4)	0 (0.0)	0 (0.0)	0 (0.0)	1 (6.7)	
**D’Amico risk groups (*n = 227*)**						**<0.001**
**Low**	81 (35.7)	45 (58.4)	30 (40.0)	6 (10.0)	0 (0.0)	
**Intermediate**	93 (41.0)	22 (28.6)	30 (40.0)	36 (60.0)	5 (33.3)	
**High**	53 (23.3)	10 (13.0)	15 (20.0)	18 (30.0)	10 (66.7)	
Post-operative characteristic						
**pT stage (*n = 230*)**						**<0.001**
**pT_1**	2 (0.9)	2 (2.5)	0 (0.0)	0 (0.0)	0 (0.0)	
**pT_2**	129 (56.1)	67 (83.8)	44 (58.7)	15 (25.0)	3 (20.0)	
**pT_3**	99 (43.0)	11 (13.8)	31 (41.3)	45 (75.0)	12 (80.0)	
**Perineural invasion in RP (*n = 214*)**						**<0.001**
**No**	43 (20.1)	26 (35.6)	11 (15.7)	6 (10.7)	0 (0.0)	
**Yes**	171 (79.9)	47 (64.4)	59 (84.3)	50 (89.3)	15 (100.0)	
**Lymph node compromise (*n = 227*)**						**<0.001**
**No**	199 (87.7)	79 (98.8)	69 (94.5)	42 (71.2)	9 (60.0)	
**Yes**	28 (12.3)	1 (1.2)	4 (5.5)	17 (28.8)	6 (40.0)	
**BCR (*n = 110*)**						**0.008**
**No**	67 (60.9)	33 (78.6)	23 (57.5)	9 (40.9)	2 (33.3)	
**Yes**	43 (39.1)	9 (21.4)	17 (42.5)	13 (59.1)	4 (66.7)	
**Additional treatment (*n = 224*)**						**<0.001**
**No**	163 (74.1)	66 (86.8)	59 (84.3)	35 (59.3)	3 (20.0)	
**ADT**	57 (25.9)	10 (13.2)	11 (15.7)	24 (40.7)	12 (80.0)	
**% tumor in RP (median, IQR)**	15.0 (7.0-30.0)	7.5 (4.0-15.0)	15.0 (10.0-27.25)	30.0 (19.5-45.0)	25.0 (18.5-55.0)	**<0.001**
**Index tumor extent - cm (median, IQR)**	1.60 (1.20-2.00)	1.15 (0.70-1.70)	1.60 (1.30-2.00)	2.00 (1.50-2.20)	2.00 (1.75-2.35)	**<0.001**
**PSA posterior to RP (median, IQR)**	0.04 (0.02-0.12)	0.04 (0.02-0.09)	0.04 (0.01-0.04)	0.08 (0.04-0.52)	0.20 (0.07-1.45)	**<0.001**
**PSA at BCR (median, IQR)**	0.26 (0.23-0.40)	0.28 (0.25-0.38)	0.25 (0.23-0.28)	0.31 (0.22-0.43)	0.54 (0.34-0.93)	**0.049**
**Time to BCR – months (median, IQR)**	17.77 (7.93-41.70)	15.73 (10.35-26.46)	39.27 (12.97-48.87)	15.88 (7.78-32.03)	7.43 (5.20-18.63)	0.084
**Time of follow-up - months (median, IQR)**	60.18 (16.43-71.93)	63.47 (16.40-75.56)	59.17 (20.70-71.93)	48.23 (10.82-70.14)	63.33 (37.62-73.48)	0.403
**European ancestry (median, IQR)**	0.55 (0.46-0.61)	0.54 (0.44-0.61)	0.54 (0.48-0.61)	0.54 (0.42-0.61)	0.60 (0.53-0.66)	0.122
**Indigenous ancestry (median, IQR)**	0.39 (0.32-0.46)	0.39 (0.33-0.47)	0.39 (0.30-0.46)	0.40 (0.34-0.47)	0.34 (0.28-0.44)	0.471
**African ancestry (median, IQR)**	0.06 (0.02-0.10)	0.07 (0.02-0.11)	0.06 (0.01-0.09)	0.07 (0.02-0.12)	0.04 (0.02-0.08)	0.5

PSA, prostate-specific antigen; RP, Radical prostatectomy.

*PSA units are ng/mL.

Bold values are statistical significant with a p-value < 0.05.

### 3.2 Population study characteristics across Gleason GG

When pathological and demographic characteristics were evaluated across the Gleason GG, higher Gleason grades were more likely to have higher initial PSA (p < 0.001), higher cT (p = 0.006), higher pathological stages at radical prostatectomies (p < 0.001), perineural invasion (p < 0.001), more cases with lymph node compromise (p < 0.001), higher percentage of tumor (p < 0.001) as well as higher index of dominant tumor nodule in radical prostatectomies (p < 0.001), and higher frequency of BCR cases (p = 0.008) (**Table 1**). Aligned with the high frequency of BCR in higher Gleason grades, these groups also presented higher values of PSA at BCR (p = 0.049), higher values of PSA after treatment (p < 0.001) and higher cases with additional treatments (p < 0.001) (**Table 1**). While not achieving statistical significance, it is noteworthy that lower Gleason grade cases exhibited a trend towards higher BMI (BMI > 25) when compared to cases with higher Gleason grades (p = 0.064) (**Table 1**).

### 3.3 Genetic ancestry structure

**Figure 1:**
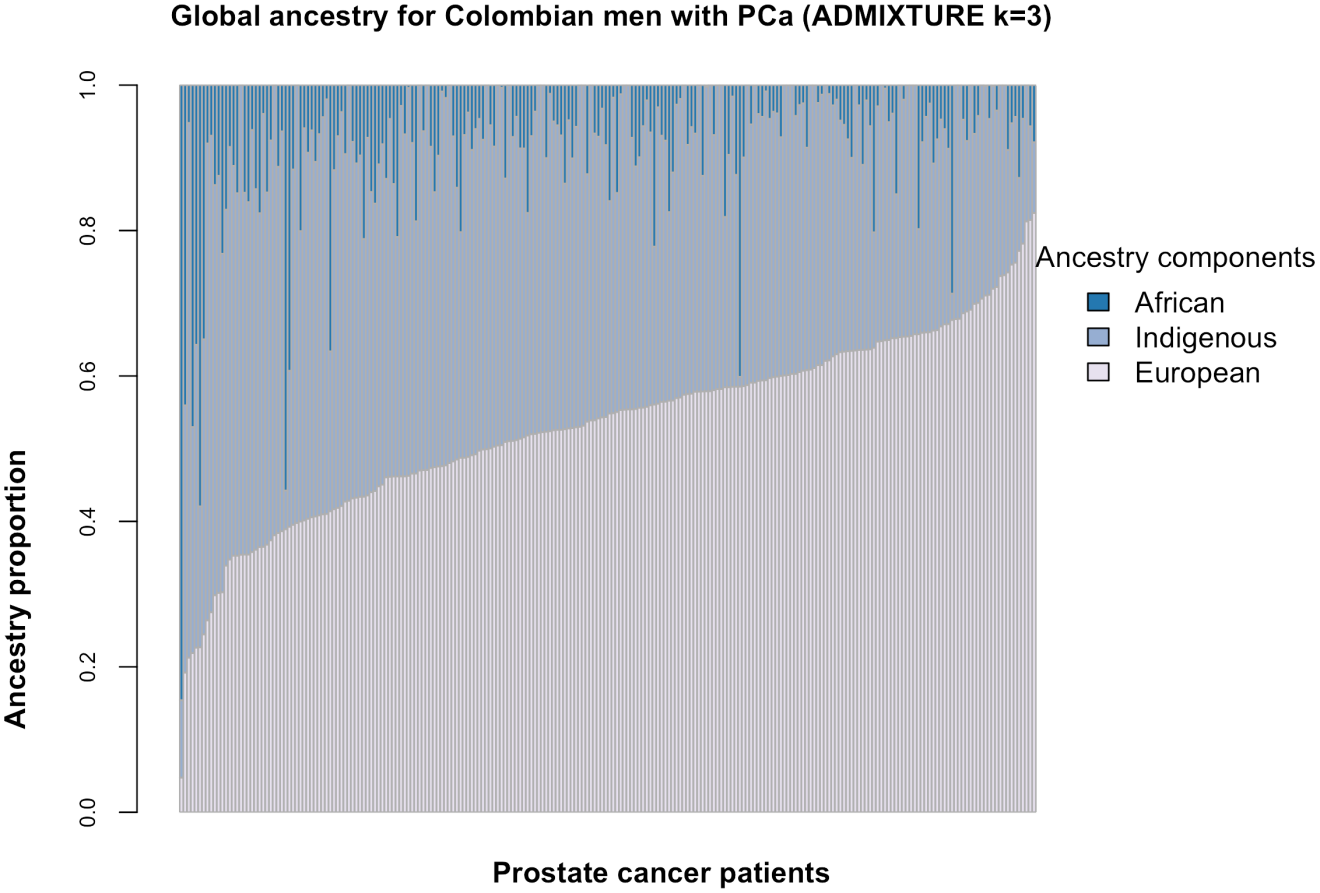
Ancestry proportion distribution per patient. Each vertical line represents an individual with each color showing the percentage of each ancestry component evaluated (k=3).

In this population, the composition of genetic ancestry showed significant variations in European and Indigenous components (**Figure 1**), with a median percentage of 55% for European ancestry and IQR of 46% and 61%, while for Indigenous ancestry the median percentage was 39% with IQR of 32% and 46%. The African component was low for this population, with a median value of 6% with IQR of 2% and 10% (**Figure 1** and **Table 1**).

### 3.4 Genetic ancestry and Gleason GG

**Figure 2:**
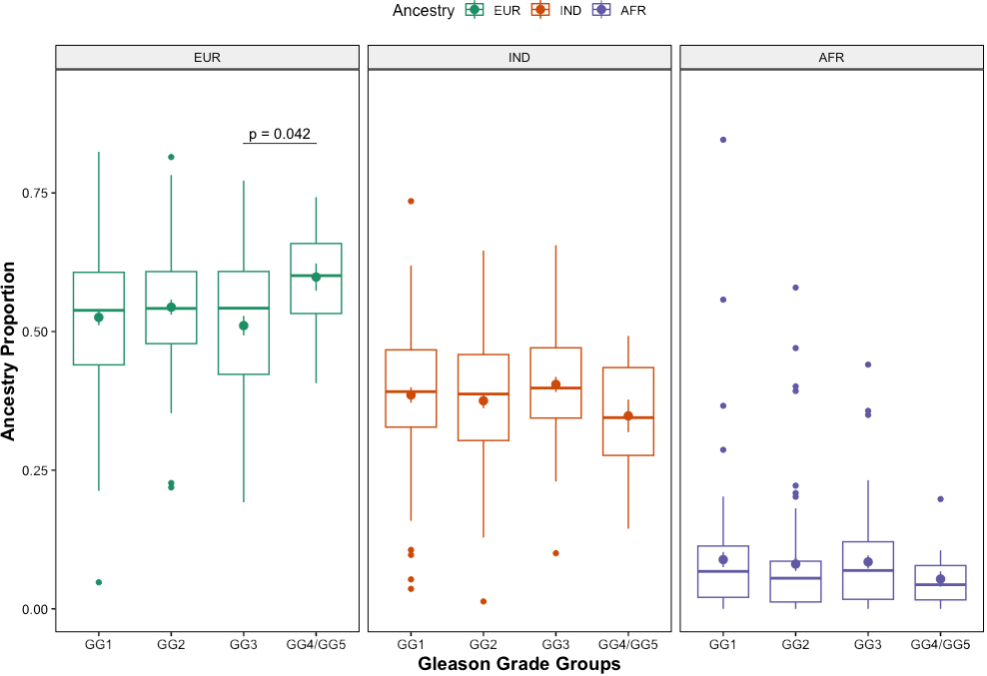
Distribution of ancestry proportions by Gleason Groups. Box plot showing the distribution of genetic ancestry proportions by Gleason Grade Group among Colombian PC patients included in the study. Two-groups comparison were made using a t-test.

The median proportion of each ancestral population (European, Indigenous and African) was calculated for each Gleason GG. Across Gleason GG1 to GG3 the median percentage of European ancestry was the same, 54%, with minor changes in the IQR between groups (44-61% for GG1, 48-61% for GG2 and 42-61% for GG3). For cases with Gleason GG4/GG5 the median European ancestry was higher, 60% and IQR between 53-66%, yet no statistically significant differences were found (**Table 1**) among all groups. However, when two group comparisons were made, there were statistically significant differences between GG3 and GG4/GG5, with GG4/GG5 showing a higher mean European ancestry proportion (p=0.042) (**Figure 2**). For Indigenous ancestry, the median proportions for Gleason GG1 and GG2 were 39% (IQR: 33-47% for GG1, 30-46% for GG2), 40% for GG3 (IQR 34-47%) and for GG4/GG5 the median proportion of Indigenous ancestry was the lowest, 34% (IQR 28-44%), although no statistically significant differences were found among groups (**Table 1**) or between two-group comparisons (**Figure 2**). Finally, for the African ancestry, the median proportions were very low in all the Gleason GG, with median values of 7% (IQR 2-11%) for GG1, 6% (IQR 1-9%) for GG2, 7% (IQR 2-12%) for GG3 and 4% (IQR 2-8%) for GG4/GG5, with no statistically significant differences across these groups (**Table 1**) or between two groups (**Figure 2**). We also considered the genetic ancestry as a dichotomous variable, we used the median value for each ancestral population as the cut-off to determine the association with the Gleason GG, however, no significant associations were found (**Table 2**). We also categorized genetic ancestry into quartiles to analyze possible differences, and we adjusted by age only and by BMI only, but no statistical findings were seen (data not shown). In addition, analyses were also done with genetic ancestry as a continuous variable to determine the variation per 25% increase in each ancestral component, still, no significant statistical associations were observed (**Table 2**).

**Table 2:** Association of genetic ancestry with Gleason Grade Groups.

Ancestry	Gleason Grade Groups			
	OR unadjusted	p value	OR adjusted^b^	p value
**Categorical variable**				
European ancestry				
**<0.546**	1.0		1.0	
**≥0.546**	1.08 (0.63-1.86)	0.78	0.97 (0.54-1.73)	0.905
Indigenous ancestry				
**<0.393**	1.0		1.0	
**≥0.393**	1.0 (0.58-1.72)	1.00	0.99 (0.55-1.76)	0.962
African ancestry				
**<0.060**	1.0		1.0	
**≥0.060**	0.74 (0.42-1.27)	0.27	0.70 (0.30-1.26)	0.237
Continuous variable				
**European ancestry**	1.95 (0.22-17.11)	0.54	1.18 (0.12-11.47)	0.884
**Indigenous ancestry**	0.90 (0.09-9.39)	0.93	1.37 (0.12-15.99)	0.802
**African ancestry**	0.45 (0.04-6.05)	0.53	0.56 (0.04-8.28)	0.66

b OR adjusted by age and BMI at diagnosis.

### 3.5 Genetic ancestry and PC characteristics

We assessed each ancestry component as a dichotomous variable to investigate its potential association with our PC patients’ demographic and clinicopathological features. As shown in **Table 3**, only patients with high African ancestry were significantly more likely to be diagnosed at an earlier median age (p = 0.02). No other significant associations were found between the clinicopathological characteristics with the genetic ancestry components.

**Table 3:** Distribution of tumor characteristics by genetic ancestry component.

Characteristics	European ancestry			Indigenous ancestry			African ancestry		
	<0.546	≥0.546	p	<0.393	≥0.393	p	<0.060	≥0.060	p
Age - years (median, IQR)	64 (42-74)	65 (32-75)	0.22	63 (42-75)	64 (32-73)	0.74	65 (32-75)	62 (42-75)	**0.02**
BMI (n = 205)			0.70			0.14			0.75
<25	37 (35.2)	33 (33.0)		32 (31.7)	38 (36.5)		36 (35.6)	34 (32.7)	
25-30	52 (49.5)	55 (55.0)		59 (58.4)	48 (46.2)		50 (49.5)	57 (54.8)	
>30	16 (15.2)	12 (12.0)		10 (9.9)	18 (17.3)		15 (14.9)	13 (12.5)	
Preoperative PSA (median, IQR)	9.21 (1.58-101.00)	8.28 (1.60-84.00)	0.28	8.44 (1.60-84.00)	8.80 (1.58-101.00)	0.35	8.40 (1.60-57.07)	8.70 (1.58-101.00)	0.80
Gleason Grade Group at biopsy (*n = 199*)			0.33			0.79			0.46
G1	66 (64.1)	55 (57.3)		59 (60.2)	62 (61.4)		60 (59.4)	61 (62.2)	
G2	21 (20.4)	16 (16.7)		17 (17.3)	20 (19.8)		16 (15.8)	21 (21.4)	
G3	12 (11.7)	18 (18.8)		15 (15.3)	15 (14.9)		18 (17.8)	12 (12.2)	
G4 and G5	4 (3.9)	7 (7.3)		7 (7.1)	4 (4.0)		7 (6.9)	4 (4.1)	
cT stage (*n = 227*)			0.22			0.92			0.28
cT_I	40 (35.4)	49 (43.0)		45 (39.8)	44 (38.6)		48 (42.5)	41 (36.0)	
cT_II	73 (64.6)	63 (55.3)		67 (59.3)	69 (60.5)		63 (55.8)	73 (64.0)	
cT_III	0 (0.0)	1 (0.9)		1 (0.9)	0 (0.0)		1 (0.9)	0 (0.0)	
cT_IV	0 (0.0)	1 (0.9)		0 (0.0)	1 (0.9)		1 (0.9)	0 (0.0)	
D’Amico risk groups (*n = 227*)			0.59			0.94			0.83
Low	39 (34.5)	42 (36.8)		39 (34.5)	42 (36.8)		42 (37.2)	39 (34.2)	
Intermediate	50 (44.2)	43 (37.7)		47 (41.6)	46 (40.4)		44 (38.9)	49 (43.0)	
High	24 (21.2)	29 (25.4)		27 (23.9)	26 (22.8)		27 (23.9)	26 (22.8)	
pT stage (*n = 230*)			0.80			1.00			0.89
pT_1	1 (0.9)	1 (0.9)		1 (0.9)	1 (0.9)		1 (0.9)	1 (0.9)	
pT_2	67 (58.3)	62 (53.9)		65 (56.5)	64 (55.7)		63 (54.8)	66 (57.4)	
pT_3	47 (40.9)	52 (45.2)		49 (42.6)	50 (43.5)		51 (44.3)	48 (41.7)	
Perineural invasion in RP (*n = 214*)			0.61			1.00			0.73
No	23 (21.7)	20 (18.5)		21 (19.6)	22 (20.6)		23 (21.5)	20 (18.7)	
Yes	83 (78.3)	88 (81.5)		86 (80.4)	85 (79.4)		84 (78.5)	87 (81.3)	
Lymph node compromise (*n = 227*)			1.00			0.31			0.57
No	100 (87.0)	99 (86.1)		102 (90.3)	97 (85.1)		97 (84.3)	102 (88.7)	
Yes	14 (12.2)	14 (12.2)		11 (9.7)	17 (14.9)		16 (13.9)	12 (10.4)	
BCR (*n = 110*)			0.25			0.44			0.44
No	31 (55.4)	36 (66.7)		35 (64.8)	32 (57.1)		37 (64.9)	30 (56.6)	
Yes	25 (44.6)	18 (33.3)		19 (35.2)	24 (42.9)		20 (35.1)	23 (43.4)	
Additional treatment (*n = 224*)			1.00			0.44			0.54
No	83 (74.1)	80 (74.1)		82 (76.6)	81 (71.7)		86 (76.1)	77 (72.0)	
ADT	29 (25.9)	28 (25.9)		25 (23.4)	32 (28.3)		27 (23.9)	30 (28.0)	
% tumor in RP (median, IQR)	15 (0-90)	15 (1-90)	0.50	15 (0-85)	15.5 (1.0-90.0)	0.78	15 (1-90)	15 (0-90)	0.39
Index tumor extent - cm (median, IQR)	1.50 (0.20-3.00)	1.70 (0.10-5.00)	0.41	1.60 (0.10-5.00)	1.60 (0.20-4.30)	0.83	1.70 (0.10-4.30)	1.50 (0.20-5.00)	0.20
PSA posterior to RP (median, IQR)	0.04 (0.00-11.40)	0.04 (0.00-38.70)	0.54	0.04 (0.00-9.09)	0.04 (0.00-38.70)	0.67	0.04 (0.00-38.70)	0.04 (0.00-11.40)	0.55
PSA at BCR (median, IQR)	0.26 (0.20-0.80)	0.26 (0.20-3.54)	0.99	0.30 (0.20-3.54)	0.25 (0.20-0.80)	0.10	0.26 (0.20-2.27)	0.29 (0.20-3.54)	0.70
Time to BCR – months (median, IQR)	22.20 (3.97-55.17)	16.04 (3.07-55.07)	0.40	15.40 (3.07-55.07)	21.72 (4.60-55.17)	0.44	18.32 (3.07-55.17)	17.77 (3.97-52.60)	0.72
Time of follow-up - months (median, IQR)	59.17 (0.93-120.83)	60.30 (0.90-116.27)	0.99	57.77 (0.90-116.27)	62.63 (0.93-120.83)	0.46	62.68 (0.90-113.97)	52.68 (0.93-120.83)	0.43

Based on Chi-square test or Fisher’s exact test for categorical variables and Kruskal-Wallis for continuous variables.

Bold values are statistical significant with a p-value < 0.05.

Interestingly, when we analyzed the associations of clinicopathological characteristics with genetic ancestry, patients with higher African ancestry had a lower risk of being diagnosed with PC at an advanced age, which means that the risk of diagnosis at a younger age increases in patients with higher African ancestry compared to lower African ancestry (OR: 0.96, CI: 0.92-0.99, p=0.03) (**Table 4**). This finding is in line with previous results described above in **Table 3**. No significant associations were found between any of the tumor characteristics and European or Indigenous ancestry (**Table 4**).

**Table 4:** Association of tumor characteristics with genetic ancestry in PC patients.

Characteristics	European ancestry		Indigenous ancestry		African ancestry	
	OR unadjusted	p value	OR unadjusted	p value	OR unadjusted	p value
Age - years (median, IQR)	1.02 (0.98-1.06)	0.32	1.00 (0.97-1.04)	0.89	0.96 (0.92- 0.99)	**0.03**
BMI (n = 205)						
<25	Ref.		Ref.		Ref.	
25-30	1.19 (0.65-2.17)	0.58	0.69 (0.37-1.25)	0.22	1.21 (0.66-2.21)	0.54
>30	0.84 (0.34-2.03)	0.70	1.52 (0.62-3.85)	0.37	0.92 (0.38-2.21)	0.85
Preoperative PSA (median, IQR)	0.99 (0.97-1.02)	0.79	1.01 (0.98-1.04)	0.50	1.01 (0.99-1.04)	0.42
Gleason Grade Group at biopsy (*n = 199*)						
G1	Ref.		Ref.		Ref.	
G2	0.91 (0.43-1.92)	0.81	1.12 (0.54-2.36)	0.76	1.29 (0.62-2.74)	0.50
G3	1.8 (0.81-4.15)	0.16	0.95(0.43-2.13)	0.90	0.66 (0.28-1.46)	0.31
G4 and G5	2.1 (0.6-8.37)	0.26	0.54 (0.14-1.90)	0.35	0.56 (0.14-1.96)	0.38
cT stage (*n = 227*)						
cT_I	Ref.		Ref.		Ref.	
cT_II	0.70 (0.41-1.20)	0.20	1.05 (0.62-1.80)	0.85	1.36 (0.79-2.33)	0.27
D’Amico risk groups (*n = 227*)						
Low	Ref.		Ref.		Ref.	
Intermediate	0.80 (0.44-1.45)	0.46	0.91 (0.50-1.65)	0.75	1.20 (0.66-2.18)	0.55
High	1.12 (0.56-2.26)	0.75	0.89 (0.45-1.79)	0.75	1.04 (0.52-2.08)	0.92
pT stage (*n = 230*)						
pT_1	Ref.		Ref.		Ref.	
pT_2	0.93 (0.04-23.73)	0.96	0.98 (0.04-25.26)	0.99	1.05 (0.04-26.87)	0.97
pT_3	1.11 (0.04-28.5)	0.94	1.02 (0.04-26.29)	0.99	0.94 (0.04-24.25)	0.97
Perineural invasion in RP (*n = 214*)						
No	Ref.		Ref.		Ref.	
Yes	1.22 (0.63-2.40)	0.56	0.94 (0.48-1.85)	0.87	1.19 (0.61-2.35)	0.61
Lymph node compromise (*n = 227*)						
No	Ref.		Ref.		Ref.	
Yes	1.01 (0.45-2.24)	0.98	1.63 (0.73-3.74)	0.24	0.71 (0.31-1.58)	0.41
BCR (*n = 110*)						
No	Ref.		Ref.		Ref.	
Yes	0.62 (0.28-1.34)	0.23	1.38 (0.64-3.01)	0.41	1.42 (0.66-3.08)	0.37
Additional treatment (*n = 224*)						
No	Ref.		Ref.		Ref.	
ADT	1.00 (0.55-1.83)	0.996	1.30 (0.71-2.39)	0.40	1.24 (0.68-2.30)	0.48
% tumor in RP (median, IQR)	1.00 (0.99-1.02)	0.42	0.99 (0.98-1.01)	0.87	0.99 (0.98-1.01)	0.35
Index tumor extent - cm (median, IQR)	1.30 (0.88 -1.96)	0.20	0.93 (0.63-1.38)	0.72	0.78 (0.52-1.15)	0.22
PSA posterior to RP (median, IQR)	1.04 (0.95-1.19)	0.46	1.08 (0.97-1.28)	0.28	0.96 (0.83-1.05)	0.43
PSA at BCR (median, IQR)	2.67 (0.79-28.42)	0.23	0.26 (0.01-1.15)	0.24	1.16 (0.39-4.20)	0.78
Time to BCR – months (median, IQR)	0.98 (0.96-1.02)	0.46	1.01 (0.98-1.04)	0.67	1.00 (0.97-1.03)	0.86
Time of follow-up - months (median, IQR)	0.99 (0.99-1.01)	0.92	1.00 (0.99-1.01)	0.39	0.99 (0.98-1.00)	0.29

Bold values are statistical significant with a p-value < 0.05.

## 4 Discussion

Our association analyses showed that the risk of being diagnosed with PC at a younger age is significantly higher in patients with high African ancestry. When we analyzed European or Indigenous ancestry no relation was found with age at diagnosis as well as with any other clinicopathological characteristics. Research has shown that age and African ancestry are independent risk factors for PC (39, 40). It has been widely described that, compared to other ethnic groups, African ancestry is associated with a higher incidence, mortality and aggressive presentation of PC (24, 41–43). And, although age is considered an independent factor, it has also been reported that African descendant population from the US and Europe may be diagnosed at a younger age compared to Caucasians, even despite equal access to health services (44–47).

Robbins et al. (45) argued that the findings from different studies with PC patients of African descent diagnosed at a younger age may be biased given the differences in age distributions among the ethnic populations with case-only comparisons, with fewer Black men than White men in older age groups (≥ 60 years old). However, even after the appropriate correction for population differences, African Americans were slightly younger than Caucasians at diagnoses for PC (1.2 years older for Caucasians compared to non-Hispanic African American men). To explain the differences in African American men, a study modeled the natural history of PC and suggested that African American men have a higher incidence of preclinical disease and are at a higher risk of metastasis than Caucasian American men (48). These findings were previously demonstrated by Powell et al. (29, 49, 50), in which they concluded that Black men are at higher risk of being diagnosed with advanced stage at a younger age and reported more frequent BCR among young Africans compared to Caucasians. These findings are important since the patients can be stratified according to their risk by screening programs, prevention, and treatment strategies. The NCCN guidelines for Prostate Cancer already suggest that men of African descent may consider starting PSA screening at a younger age, typically around 40 to 49 years old (51). Furthermore, draws important attention to investigating the etiology of the disease in these patients with early onset presentation.

The studies above mentioned were mainly based on African descendants and Caucasian populations from the United States and Europe, and they did not include Hispanic/Latino populations in their analyses. It was previously reported that the survival in Hispanics is better compared to Black men and White men (52); however, it is well known that the Hispanic/Latino population is highly heterogeneous, descending from generations of admixing of Indigenous Latin American, European and African populations, as well as bearing various degrees of admixture across Latin American countries (11). Among Hispanic populations in the US, it was reported that the incidence of PC differs by country/region of origin or genetic ancestry (53). A recent study in the US analyzing Hispanic/Latino subgroups (54), with local-regional PC, showed that Puerto Rican men a have greater risk of PC-specific mortality compared not only to non-Hispanic White men but also to non-Hispanic Black men in the US, while Mexican patients had similar risk to non-Hispanic Black men although higher than non-Hispanic White men. These findings highlight the emphasis that should be made for a deeper understanding of the etiology of the disparities embracing Hispanic/Latino populations in PC. Given the nature of our study, we could not have data on PC mortality for these patients, instead, we captured BCR information. However, no association with African ancestry or any other ancestry groups was found. This could be an effect of the small sample size of patients with BCR information. However, it also could be explained by the limitations that we describe further.

We calculated the proportions of European, Indigenous and African ancestries for each patient under an admixture model based on the genotyping of 106 Ancestry-Informative Markers. The median composition of our cohort was 55% European, 39% Indigenous and 6% African. Different results in a cohort of Hispanic/Latino population of Puerto Rican patients with PC were determined by Berglund et al. (55) with an average of 65.8% for the European component, 21.9% for the African component and 12.3% for the Indigenous American component, yet the same methodology to estimate genetic ancestry was used. Studies in Colombian cohorts for other types of cancer found also slightly different results to ours with an approximate mean composition of 56% European, 31% Indigenous and 10% African for colorectal cancer (35) and 53% European, 38% Indigenous and 9% African for breast cancer (56). Finally, a recent study reported different genetic ancestry compositions for two cohorts in Colombia (57): the first one composed of 624 individuals, reported an average European ancestry of 55%, Native American ancestry of 32.4% and African ancestry of 12.6%; slightly similar to our results. The second cohort was composed of 99 individuals from another geographically different region in the country, characterized for being mostly an Afro-Colombian ethnic region, with an average European ancestry of 11.5%, Native American ancestry of 12.5% and African ancestry of 76%. Moreover, they reported a positive correlation between the genetic African ancestry and the predictive risk of PC for the Colombian population (57). Dissimilarities in the components of genetic ancestry across Hispanic/Latino countries and even within Colombian populations, reinforce the high levels of genetic admixture in various degrees, as we described above, and that PC may have both a strong biological basis (8) as well as a socioeconomic determinant (6, 53). Yet, this is the first study analyzing the relationship between the genetic ancestry with the clinicopathologic characteristic in Colombian patients with PC.

The distribution of Gleason GG found in our patients is variable compared to other studies with patients bearing localized PC tumors. While Zimmermann et al. (58) reported 12% of cases in GG1 and 29% for GG4/5, Chien et al. (59) reported 35% of the cases in GG1, which resembles the distribution in our study with 34.8% of cases in GG1. However, Chien et al. also reported 20% of cases in GG4/5, differing from our 6.5% for the same group. For a Hispanic/Latino population, a recent study with Puerto Rican patients (55) showed 35% of their cases in higher Gleason GG (GG3, GG4 and GG5 as converted from the Gleason patterns), similar to our results, with 32.6% of our cases in GG3 and GG4/GG5. Dissimilarities across studies may be given by some differences in the inclusion criteria, including positive margins in the Zimmermann study, which could enrich their cases in higher Gleason GG; in contrast, for our study only patients with localized/regionally advanced PC with RP surgery as treatment were included, which may explain the distribution in the frequencies of Gleason GG with lower cases in GG4/GG5.

The grading system for the classification of patients, by the International Society of Urological Pathology in 2014, is a significant independent predictor of both biochemical recurrence and clinical recurrence after RP (60). In our previous study, we reported that Gleason GG2 and GG4/5 at RP were associated with the risk of BCR, compared to GG1 (p=0.037 and p=0.08, respectively) (38). We observed here that higher Gleason GG were associated with worse pathology and clinical features, as well as more cases having BCR within 5 years after RP. These data are supported by previous evidence showing the prognostic value of Gleason for BCR and metastatic disease in US (61) and Asian (62, 63) populations. Moreover, a study in Afro-Caribbean patients not only found Gleason GG as a predictor for BCR-free survival and disease-free survival, in addition, they reported that Gleason GG outperformed the D’Amico classification to distinguish risk (64).

When we analyzed the relation between BMI across Gleason GG at RP, we noticed that most of the cases were overweight and obese (BMI ≥25: 65.9%, n=135) resembling the report by Zimmermann et al. (58) with 59% of their cases diagnosed within the same group (BMI ≥25); yet, these results differ from those reported by Woo et al. (65), with a 61.6% of their cases diagnosed with a BMI <25. Within the highest Gleason GG4/GG5, most of our cases were diagnosed at normal BMI, with a lower frequency of overweight and obese cases compared to GG1 to GG3. Although BMI across Gleason GG did not reach statistical significance (p = 0.064), this is consistent with a previous report which found significantly higher Gleason score and increased risk of BCR in normal-weight men compared to overweight and obese (66); however, our results differ from most of the studies conducted in Western populations reporting higher incidence of PC and more aggressive disease in obese man compared to normal (67–70). We must acknowledge that we made our analyses based on Gleason GG at RP and not using Gleason GG at biopsy, as most studies do. Nevertheless, the proposals and updates of the grading system throughout the years have been verified in patients treated with RP given the availability of the entire sample, and thus a more precise correlation of the Gleason grading system with the prognosis of patients, although biopsies were also analyzed for validation (15, 16, 19, 60). The distribution of BMI in our cases may also be influenced by the sample size and low representation of higher Gleason GG given by the inclusion criteria for our patients with localized PC, described ahead in the limitations. Moreover, we have to consider that BMI does not discriminate between muscle and fat content and it does not take into account fat distribution patterns (71). The differences among studies may reflect the fact that BMI is not as accurate as other measurements to associate with the risk or aggressiveness of PC, as proved widely previously (65, 72). It is becoming clear that obesity is associated with a greater risk of high-grade prostate cancer, despite the several confounding factors, such as the effect of obesity on cancer detection, treatment decisions, treatment efficacy, and treatment complications (73, 74).

Compared to other Latino populations, the PSA values at diagnosis (8.65 ng/mL, IQR 6.21 – 13.49 ng/mL) were slightly higher in our cases while Murray et al. (75) reported PSA median values of 5.5 ng/mL (IQR 3.34) for Chilean patients treated for RP. However, the PSA values at diagnosis in our study are similar to a study in a Brazilian population with a median PSA value of 8 (IQR 0.28 – 51 ng/mL).

Our study has inherent limitations. First, this is a hospital-based study, and even though the INC is the main cancer center in the country, it may not be fully representative of the country’s population since patients were mainly from the Andean region with limited involvement of patients from the Coastal regions. This may explain the low fraction of the African ancestry in most of our patients and limited our ability to identify deep associations with the clinicopathological features of our cohort. Second, the retrospective design and short follow-up limited us from having other outcomes, as well as from gather information of self-reported ethnicity to explore the social determinants in the aggressiveness of the disease through possible correlations. Third, a high number of patients at the INC encounter significant health access barriers during their treatment and follow-up. These barriers include transportation challenges and a fragmented healthcare system. This fragmentation can lead to delays in clinical interventions when patients transition between different health insurers and cancer institutes. Fourth, the small sample size and the inclusion criteria of patients, which focused on localized or regionally advanced PC who underwent RP [and with available tumor and non-tumor tissue samples for the study (38)], might explain the low representation of higher Gleason GG and aggressive cases and may have limited the associations found in the analyses.

## 5 Conclusions

We have analyzed a cohort of PC Hispanic/Latino men treated with RP evaluating whether genetic ancestry in Colombian patients plays a role in the aggressiveness of PC. This is the first study to comprehensively assess the associations of genetic ancestry with PC in the Colombian population. There was no association between genetic ancestry with BCR. However, the results showed an increased risk for the onset of PC at a younger age for patients with higher African ancestry, which can have an impact on screening and management of the disease, as well as indagate the etiology of the disease.

## Data availability statement

The raw data supporting the conclusions of this article will be made available by the authors, without undue reservation.

## Ethics statement

The studies involving humans were approved by Comité de Ética en Investigaciones (CEI) from Instituto Nacional de Cancerología in Colombia. The studies were conducted in accordance with the local legislation and institutional requirements. The human samples used in this study were acquired from a by- product of routine care or industry. Written informed consent for participation was not required from the participants or the participants’ legal guardians/next of kin in accordance with the national legislation and institutional requirements.

## Author contributions

NA: Conceptualization, Data curation, Formal analysis, Investigation, Methodology, Writing – original draft. RV: Conceptualization, Data curation, Writing – review & editing. JM: Conceptualization, Data curation, Writing – review & editing. JG: Data curation, Methodology, Writing – review & editing. AG: Conceptualization, Supervision, Writing – review & editing. SS: Writing – review & editing, Formal analysis, Methodology. JZ: Conceptualization, Data curation, Formal analysis, Funding acquisition, Methodology, Resources, Supervision, Writing – review & editing. MS: Conceptualization, Data curation, Formal analysis, Funding acquisition, Methodology, Resources, Supervision, Writing – review & editing. AC: Conceptualization, Data curation, Formal analysis, Funding acquisition, Methodology, Resources, Supervision, Writing – review & editing.
